# Supplementary figures and images for: A Comparative Study of Engineered Dermal Templates for Skin Wound Repair in a Mouse Model
Source: Int J Mol Sci. 2020 Jun 25;21(12):4508. doi: 10.3390/ijms21124508 (PMC7350005; doi:10.3390/ijms21124508)

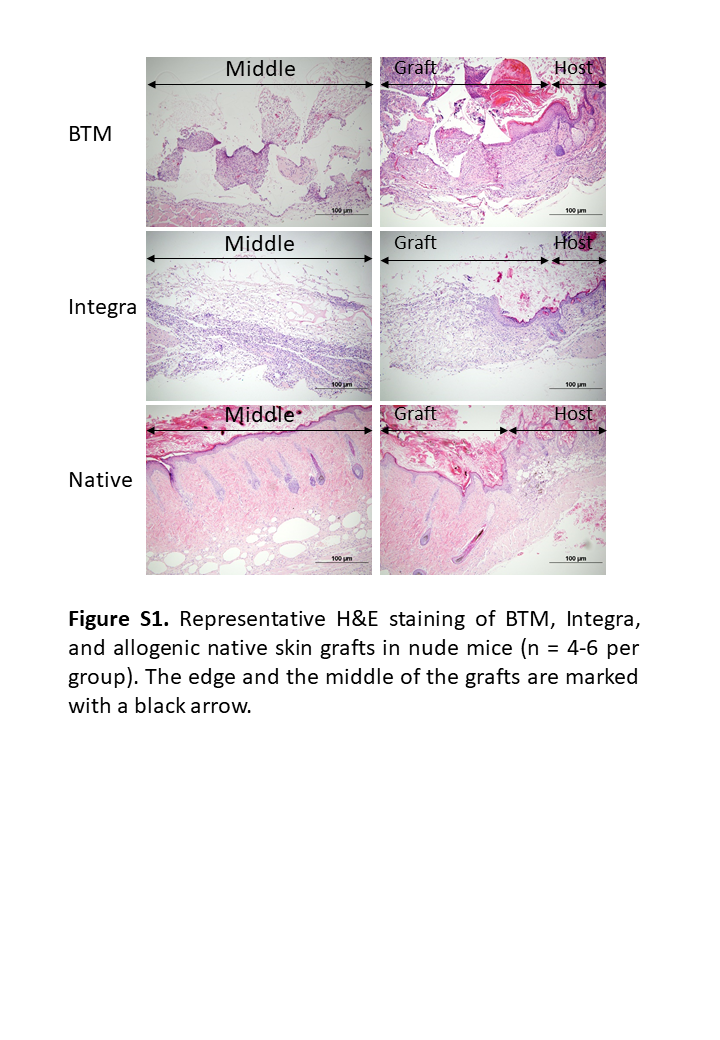

Supplement: Supplementary file 1 [file ijms-21-04508-s001.zip › Slide1.TIF]

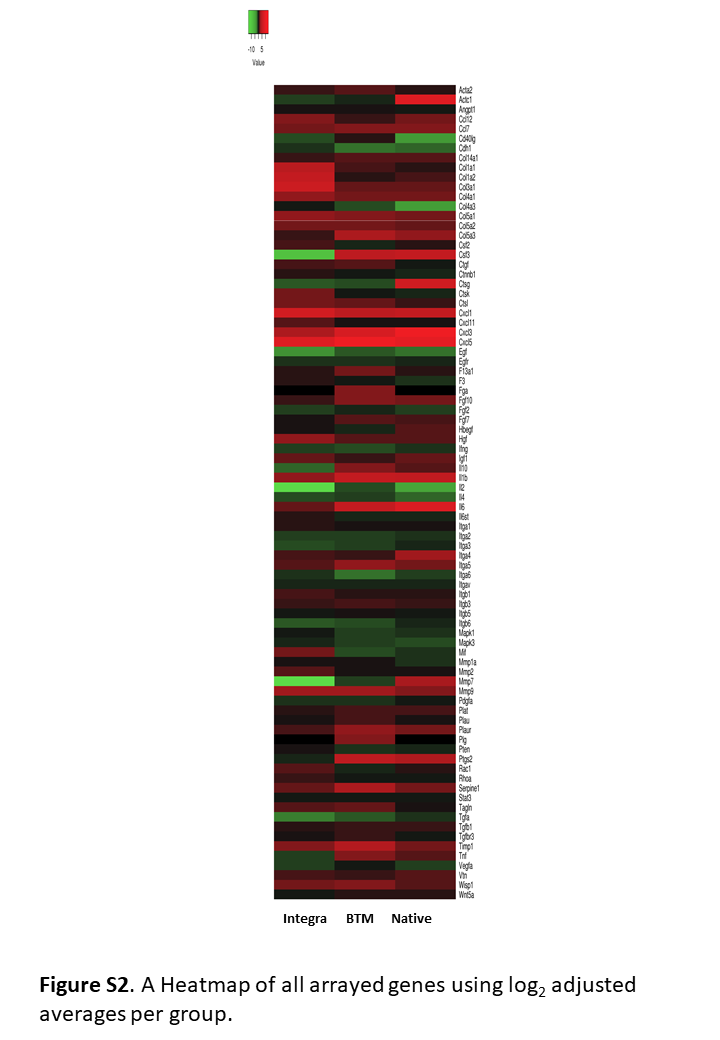

Supplement: Supplementary file 1 [file ijms-21-04508-s001.zip › Slide2.TIF]

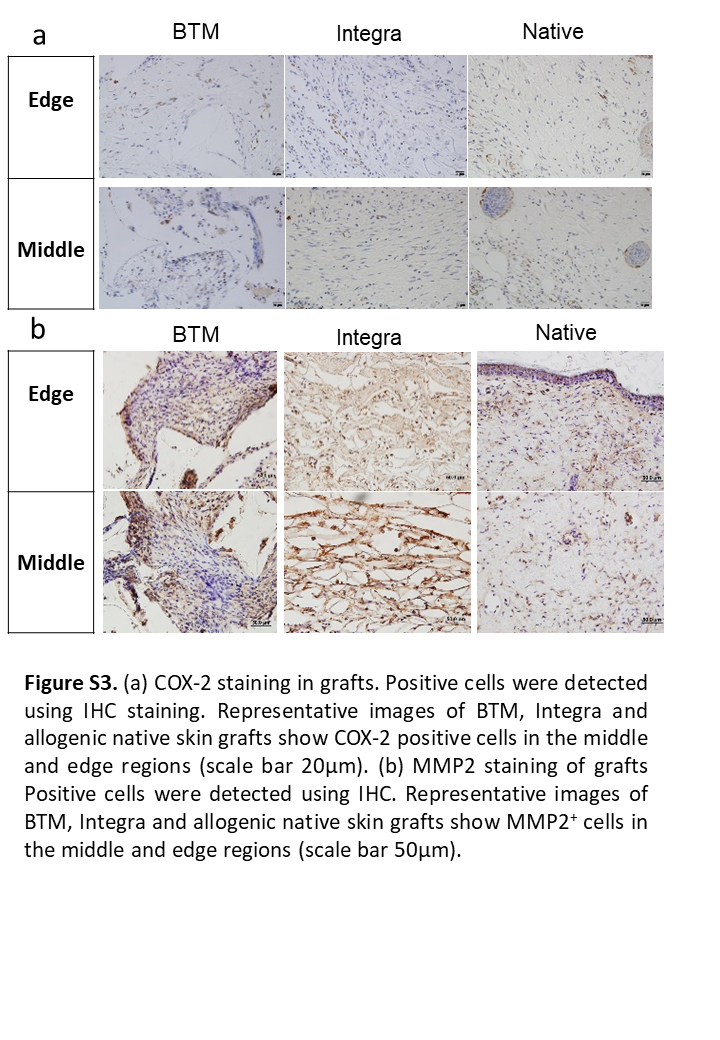

Supplement: Supplementary file 1 [file ijms-21-04508-s001.zip › Slide3.TIF]

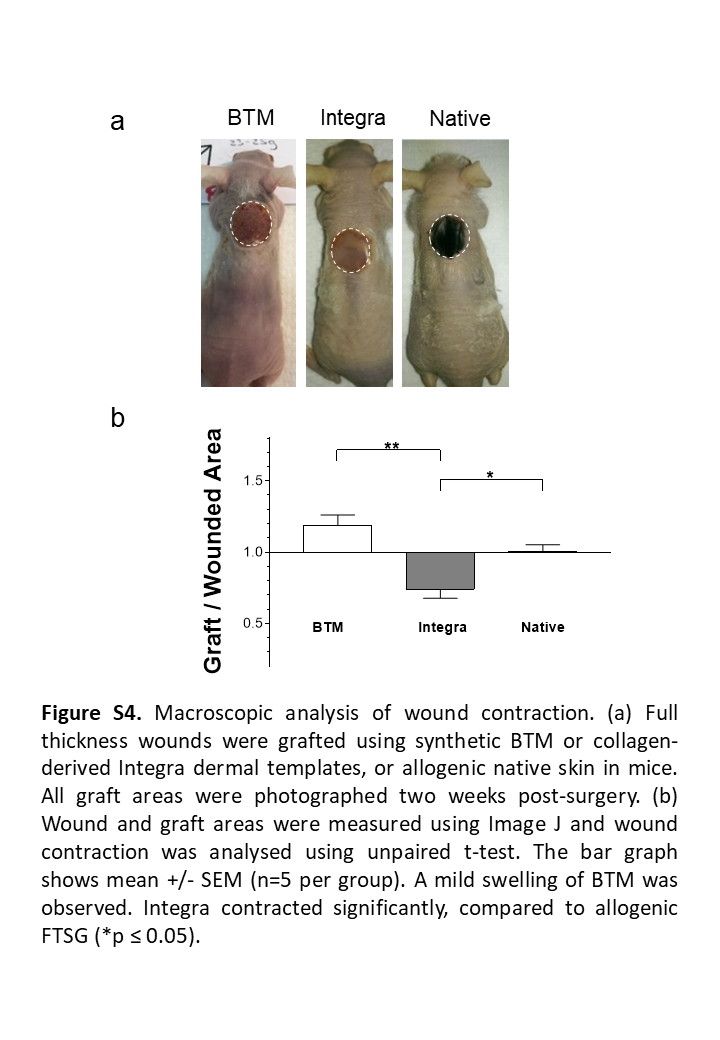

Supplement: Supplementary file 1 [file ijms-21-04508-s001.zip › Slide4.TIF]

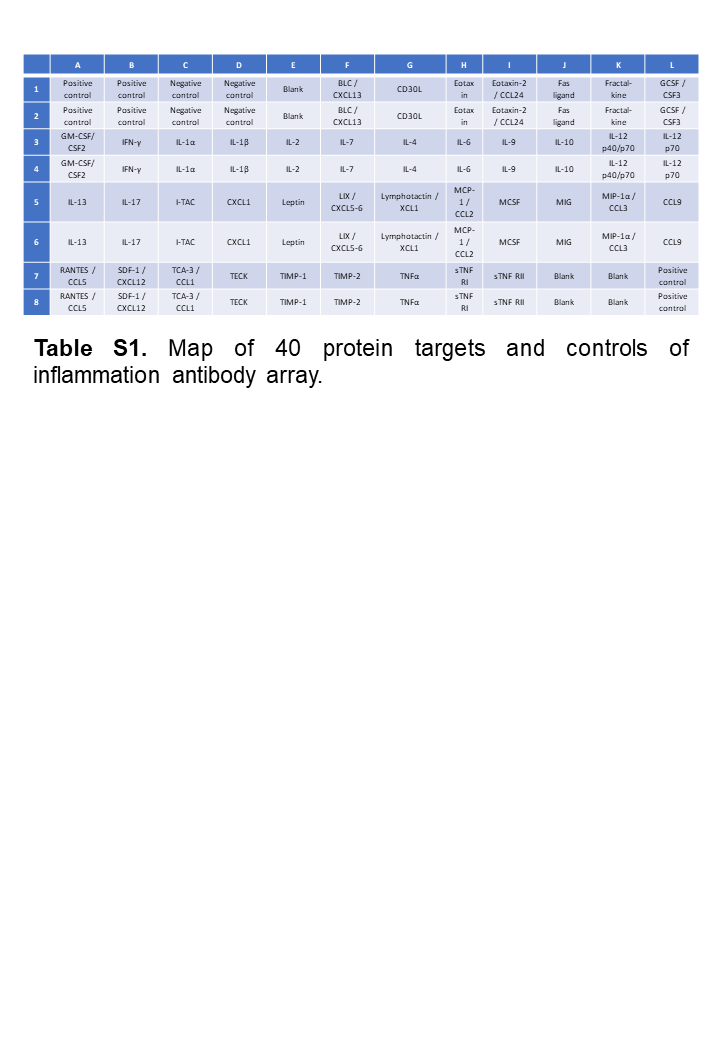

Supplement: Supplementary file 1 [file ijms-21-04508-s001.zip › Slide5.TIF]
